# Timing matters: there are significant differences in short-term outcomes between two time points of status epilepticus

**DOI:** 10.1186/s12883-022-02868-y

**Published:** 2022-09-14

**Authors:** Yiwen Pan, Yu Feng, Weifeng Peng, Yang Cai, Jing Ding, Xin Wang

**Affiliations:** 1 Department of Neurology, Zhongshan Hospital, Fudan University, 180 Fenglin Road, Xuhui District, Shanghai, 200032 China; 2 CAS Center for Excellence in Brain Science and Intelligence Technology, Shanghai, 200032 China; 3 The State Key Laboratory of Medical Neurobiology and MOE Frontiers Center for Brain Science, The Institutes of Brain Science and the Collaborative Innovation Center for Brain Science, Fudan University, Shanghai, 200032 China

**Keywords:** Status epilepticus, Seizure duration, Mortality, Seizure recurrence, Functional status

## Abstract

**Background:**

In 2015, the International League Against Epilepsy proposed a new conceptual definition of status epilepticus (SE) with two operational dimensions (t1 and t2) to guide emergency treatment. The purpose of this study was to compare clinical characteristics and prognoses of patients at these two different time points.

**Methods:**

We conducted a prospective observational cohort study of consecutive adults diagnosed with SE. In case of convulsive SE, t1 is 5 min and t2 is 30 min, whereas in case of focal SE with impaired consciousness, t1 is 10 min, t2 is 60 min. Data on clinical characteristics, including age, gender, history of prior seizures, neuroimaging, semiology, duration, and etiology of SE, were collected. The primary outcome was mortality, with seizure recurrence as a secondary measure, and functional status as tertiary outcome of enrolled patients at 3 months after SE onset.

**Results:**

We screened one hundred patients with SE, with a median age of 66 years and 61% were male. Fifty-six (56.0%) patients reached t1 of SE, while 44 (44.0%) reached t2 of SE. Convulsive SE (52.0%, *n* = 52) was more common than focal SE with impaired consciousness (48.0%, *n* = 48). Status epilepticus secondary to an acute symptomatic process was the most common (50%, *n* = 50). Patients meeting t2 of SE demonstrated a remarkably increased risk of mortality (unadjusted analysis-RR 3.606, 95%CI 1.552–8.376, *p* = 0.003; adjusted analysis-RR 2.924, 95%CI 1.221–7.003, *p* = 0.016) and unfavorable functional status (unadjusted analysis-RR 1.803, 95%CI 1.280–2.539, *p* = 0.001; adjusted analysis-RR 1.664, 95%CI 1.184–2.340, *p* = 0.003) at 3 months compared to those who only reached t1 of SE. Patients reaching t2 of SE were more likely to experience seizure recurrence, however, there was no significant difference between the two cohorts.

**Conclusions:**

Our study provides strong support for the new definition of SE. Patients meeting t2 of SE tend to have a remarkably increased risk of mortality and unfavorable functional outcomes compared to those who only reached t1 of SE. Furthermore, patients were likely to experience seizure recurrence after undergoing an episode of SE. Physicians must be educated about prompt recognition and appropriate management of SE.

**Supplementary Information:**

The online version contains supplementary material available at 10.1186/s12883-022-02868-y.

## Introduction

Status epilepticus (SE) is a life-threatening neurological emergency. Based on previous epidemiological data, SE has an annual incidence of 10–41 per 100,000 people [1]. The overall mortality for SE varies from 1.9 to 40% and depends mainly on age (over 65 years > 20–64 years > below 20 years), etiology, and seizure duration. Anoxia and hypoxia are associated with the highest mortality, followed by stroke, central nervous system (CNS) infections, and metabolic disorders [2–7]. Evidence suggests that seizures lasting more than 30 min have a significantly higher mortality rate than seizures lasting 10–29 min [8]. Furthermore, seizure duration is the only modifiable risk factor that can be improved by rapid intervention. Therefore, it is essential to precisely define the seizure duration in SE for enhanced clinical decision making.

The classical definition with a 30-min cut-off of SE was put forward by the American Epilepsy Society in 1993 [9, 10]. It reflects the loss of auto-regulatory mechanisms, metabolic decompensation, and often irreversible neuronal damage that occurs with prolonged convulsive seizures, as demonstrated in previously healthy primate models [11]. However, based on observational studies, most generalized tonic–clonic seizures are unlikely to last more than 2–3 min before resolving spontaneous [12, 13]. A recent study also showed that the cumulative clinical seizure duration (99%) was 7 min in focal impaired awareness seizures and 11 min in focal aware seizures with motor symptoms [14]. A seizure lasting longer than the average duration is unlikely to terminate spontaneously and can have progressive resistance to benzodiazepines (BZDs). Moreover, evidence from animal data on neuronal injury and pharmacoresistance indicated that it is unreasonable to wait for treatment [15]. Taken together, these findings led the International League Against Epilepsy (ILAE) to reach a consensus that treatment for convulsive seizures should begin in approximately 5 min.

In 2015, the ILAE proposed a new conceptual definition of SE with two operational dimensions to provide a framework for clinical diagnosis, investigation, and therapeutic approaches for each patient. The first is the length of the seizure and the time point (t1) beyond which the seizure should be regarded as an “abnormally prolonged seizure.” The second is the time point (t2) of ongoing seizure activity, after which there is a risk of long-term consequences, including neuronal death, neuronal injury, and alteration of neuronal networks [16]. In the case of convulsive SE, t1 is 5 min and t2 is 30 min, which is based on animal experiments and clinical research. In focal SE with impaired consciousness, t1 is 10 min while t2 is more than 60 min. Nevertheless, the evidence is incomplete, and there is considerable variation. Data on other forms of SE are limited.

To date, there are no clinical studies regarding the two operational dimensions of SE in either prehospital or inhospital settings. We performed a prospective observational cohort study with two aims. The first was to analyze demographic characteristics, seizure semiology, and etiological risk factors of patients with SE based on the new definition proposed by the ILAE at the two different time points. The second was to identify whether these two different time points in patients with SE lead to different prognoses, including mortality, seizure recurrence, and functional status.

## Methods

### Study design

A prospective cohort study at Zhongshan Hospital, a tertiary academic medical institution in Shanghai, China, was performed. Consecutive adult patients with SE, from June 1, 2017, to December 31, 2018, were recruited for this study. This cohort included patients admitted for SE and also patients developing SE during the hospital stay, but those associated with acute postanoxic encephalopathy were excluded due to the high rate of mortality [2]. The diagnosis of SE was derived from the 2012 Neurocritical Care Society Guidelines [17], SE was defined as continuous clinical or electrographic seizure activity or as recurrent seizure activity without interictal recovery. The SE duration time points were based on the 2015 ILAE guidelines. In the case of tonic–clonic SE, t1 is 5 min while t2 is 30 min. In focal SE with impaired consciousness, t1 is 10 min while t2 is more than 60 min. As for absence SE, t1 is 10–15 min, and t2 is unknown [16].

### Patients and data collection

Because this study focused on the acute phase of critically ill patients, written informed consent was obtained from participants’ immediate family members. The study was approved by the ethics committee of Zhongshan Hospital and have been performed in accordance with the ethical standards as laid down in the 1964 Declaration of Helsinki and its later amendments. The eligibility criteria were as follows: (1) individuals diagnosed with and managed for SE; (2) age 18 years or older; (3) admission between June 1, 2017, and December 31, 2018; (4) tonic–clonic SE or focal SE with impaired consciousness, as time points are not yet available for other forms of SE based on the 2015 ILAE guidelines. In cases in which a patient had more than one SE episode during the study period, only the first episode was entered into the study.

Exclusion criteria were as follows: (1) nonepileptic seizures, including cardiogenic and neurogenic syncope, psychogenic nonepileptic seizure, transient ischemic attack, and panic attack; (2) nonconvulsive SE (NCSE) detected on electroencephalogram (EEG) without prominent motor symptoms; (3) unknown time of seizure duration.

Collected clinical variables included age, gender, history of prior seizures, neuroimaging, SE semiology (convulsive or nonconvulsive, generalized or focal), seizure duration, and etiology of SE. For prehospital SE, seizure duration time were prospectively obtained from patients’ families and confirmed with ambulance reports and medical documents. As for inhospital SE, information was acquired from medical records and attending physicians. In each case, the seizure duration time was estimated as the time from symptoms onset to an absence of clinically apparent seizures [18], and we excluded patients if the duration time was unclear. Clinically apparent seizures were determined by eyewitnesses and were defined as visually observed facial or body movements. SE etiology was categorized according to the guidelines of the ILAE into acute symptomatic, remote symptomatic, progressive symptomatic, and unknown etiology [16]. Additional specific etiologies were also ascertained, which referred to the etiology section of the Epidemiology-based Mortality Score in Status Epilepticus (EMSE) scale [19]. Furthermore, a potentially fatal etiology (PFE) was defined when meeting the criteria introduced by previous literature [20].

For neuroimaging, computed tomography (CT) or magnetic resonance imaging (MRI) after SE onset were collected and classified into three categories: no responsible lesion, unilateral responsible lesions, and bilateral responsible lesions or diffuse cerebral edema [21].

After admission, within 72 hours of seizure onset, each patient underwent bedside video-EEG (10/20 international electrode system) monitoring for at least 2 hours to detect special patterns [22] and guide treatment. Meanwhile EEG was indispensable for the final diagnosis of NCSE [23, 24].

Participants were dichotomized into two cohorts based on the new definition of SE: (1) patients only meeting the first time point and (2) patients reaching the second time point. Clinical data were authenticated by two trained neurologists. All enrolled patients were followed up for at least 3 months after SE onset. Information on outcome was extracted from telephone calls or medical records if patients had represented to our hospital.

The primary outcome was mortality, with seizure recurrence as a secondary measure at 3 months after SE onset. We also used the modified Rankin Scale (mRS) to measure functional status of enrolled patients as tertiary outcome. This scale comprises seven different levels of outcomes, ranging from 0 (no symptoms) to 5 (severe disability) and 6 (death) [25]. For the purpose of statistical analysis, we defined a score range of 0–2 as a favorable outcome, while a score range of 3–6 was considered an unfavorable outcome.

### Statistical analysis

Statistical analysis was performed using SPSS version 22.0. All tests were two-sided, and a *p* value of less than 0.05, was considered statistically significant. Continuous variables were expressed as mean and standard deviation (normally distributed), as median and interquartile ranges ([IQR], not normally distributed), or as counts and percentages if they were categorical variables. Baseline demographic data and clinical characteristics were compared using the Pearson chi-square test or Fisher’s exact test for categorical data and the Mann–Whitney U test or Student’s t-test for continuous variables.

We first performed univariate analysis for each outcome using the Pearson chi-square test or Fisher’s exact test for the categorical variables and the Mann–Whitney U test or Student’s t-test for the continuous variables. To evaluate the association of different time points of SE with each outcome, we then conducted modified Poisson regression [26] without and with adjustment for any covariate with univariate significance of a *p*-value less than 0.05 (the variables included in it are age, gender, history of prior seizures, acute symptomatic etiology, potentially fatal etiology, bilateral lesions/diffuse cerebral edema and interictal epileptiform activity) to allow estimation of relative risk. These calculations were performed on the overall cohort as well as subgroups of different SE semiology (tonic–clonic SE and focal SE with impaired consciousness).

## Results

### Demography and clinical characteristics

**Fig. 1:**
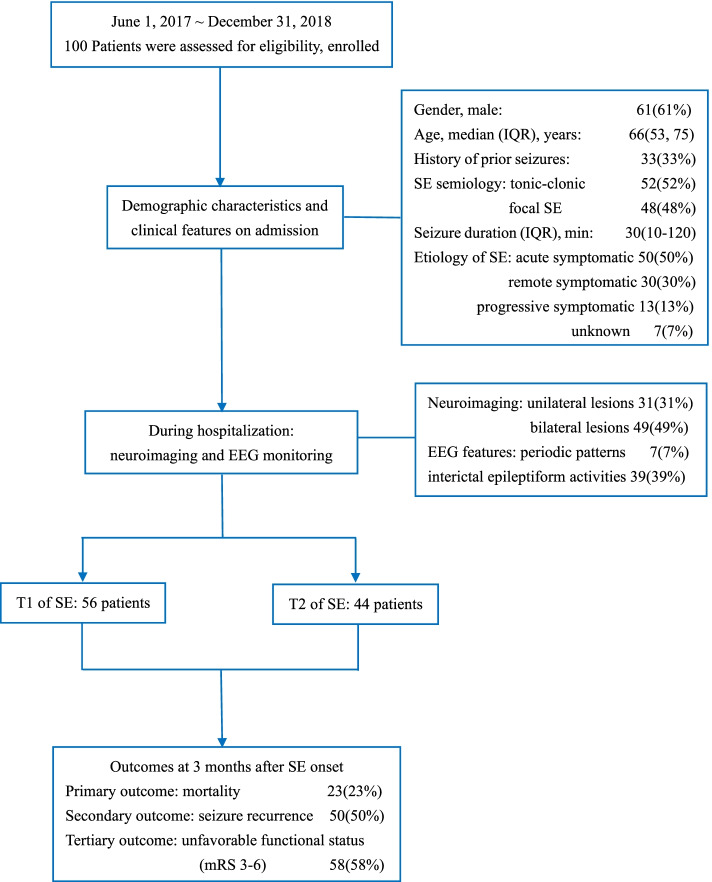
Flowchart of study cohort. One hundred patients were enrolled for final analysis between June 1, 2017, and December 31, 2018. Fifty-six (56.0%) patients reached t1 of SE, while 44 (44.0%) reached t2 of SE. Clinical characteristics and prognoses of patients were presented briefly. mRS: modified Rankin Scale, SE: status epilepticus

The patient flow chart is shown in Fig. 1. One hundred subjects were identified who fulfilled inclusion criteria, each of whom was followed up. The median age of the cohort was 66 (IQR, 53–75) years, and 61.0% (*n* = 61) were male. Fifty-six (56%) patients met the first time point of the 2015 ILAE guideline’s SE definition but did not reach the second time point, while forty-four (44.0%) reached the second time point. Premorbid seizures occurred in 33.0% of patients. Fifty-two subjects had tonic-clonic SE (including both generalized convulsive SE and focal onset evolving into bilateral convulsive SE), followed by forty-eight with focal SE with impaired consciousness. EEG monitoring showed ictal discharges in 19 patients (19.0%), interictal epileptiform activities in 39 (39.0%) and periodic patterns in 7 (7.0%). The demographic manifestations, clinical, neuroimaging and EEG features are summarized in Table 1.

**Table 1:** Baseline demographic characteristics and clinical features

	Total cohort (*N* = 100)	T1 of SE (*n* = 56)	T2 of SE (*n* = 44)	*P* value
Demographics				
Gender, male	61(61.0%)	37(66.1%)	24(54.5%)	0.303
Age, median (IQR), years	66(53, 75)	64.5(46.8, 73,8)	67(56, 81.8)	0.133
History of prior seizures	33(33.0%)	20(35.7%)	13(29.5%)	0.530
SE semiology				0.314
Tonic-clonic SE	52(52.0%)	32(57.1%)	20(45.5%)	
Focal SE with impaired consciousness	48(48.0%)	24(42.9%)	24(54.5%)	
Seizure duration (IQR), min	30(10–120)	10(10–15)	174.9(100.2–540)	<0.001 ***
Etiology of SE				
Acute symptomatic	50(50.0%)	24(42.9%)	26(59.1%)	0.158
Remote symptomatic	30(30.0%)	19(33.9%)	11(25.0%)	0.384
Progressive symptomatic	13(13.0%)	7(12.5%)	6(13.6%)	0.999
Unknown	7(7.0%)	6(10.7%)	1(2.3%)	0.131
Neuroimaging				
unilateral lesions	31(31.0%)	18(32.1%)	13(29.5%)	0.830
bilateral lesions/diffuse cerebral edema	49(49.0%)	23(41.1%)	26(59.1%)	0.107
EEG features				
Interictal epileptiform activities	39(39.0%)	18(32.1%)	21(47.7%)	0.149
Periodic patterns	7(7.0%)	4(7.1%)	3(6.8%)	0.999

*EEG* Electroencephalogram, *IQR* Interquartile range, *SE* Status epilepticus

***p<0.001

SE secondary to an acute symptomatic process was the most common, accounting for 50.0% (n = 50) of the cases, followed by remote symptomatic process (30.0%, *n* = 30), progressive symptomatic process (13.0%, *n* = 13), and unknown process (7.0%, *n* = 7) (Table 1).

In addition, the most common causes of SE were remote cerebrovascular disease, brain injury (20.0%, *n* = 20), acute cerebrovascular disease (19.0%, *n* = 19), drug reduction/withdraw, poor compliance (17%, *n* = 17) and acute CNS infection (16%, *n* = 16). Fifty-one patients (51.0%) had a potentially fatal etiology (PFE). A description of the specific causes of SE is presented in Table 2.

**Table 2:** Specific causes of SE

Cause	T1 of SE (*n* = 56)	T2 of SE (*n* = 44)	*P* value
Drug reduction/withdraw, poor compliance	13(23.2%)	4(9.1%)	0.106
Remote cerebrovascular disease, brain injury	12(21.4%)	8(18.2%)	0.803
Head trauma	1(1.8%)	3(6.8%)	0.317
Brain tumor	6(10.7%)	5(11.4%)	0.999
Metabolic disorders	7(12.5%)	2(4.5%)	0.292
Acute cerebrovascular disease	6(10.7%)	13(29.5%)	0.022*
CNS-infection: acute	8(14.3%)	8(18.2%)	0.784
Cryptogenic	3(5.4%)	1(2.3%)	0.629
Potentially fatal etiology (PFE)	23(41.1%)	28(63.6%)	0.029*

*CNS* Central nervous system, *SE* Status epilepticus

*p<0.05

### Factors associated with SE timing

Age, gender, history of prior seizures, SE semiology, neuroimaging and EEG features did not differ significantly between the two cohorts with SE at different time points. Episodes of SE reaching the second time point were more common for the acute symptomatic process, however the difference was not significant (Table 1).

Analysis of SE timing regarding specific causes demonstrated significant differences in acute cerebrovascular disease and a potentially fatal etiology. In other words, patients with acute cerebrovascular disease or had a PFE were more likely to meet the criteria of the second time point of SE (Table 2).

Seizures lasted for a significantly shorter time in patients who reached t1 of SE (median time: 10 min; IQR: 10 min, 15 min) than in those who reached t2 (median time: 174.9 min; IQR: 100.2 min, 540 min). The cause of seizure termination was evaluated at the two time points of SE. Among the patients who reached t2 of SE, 90.9% (*n* = 40) required intravenous antiepileptic drugs to terminate SE, and very few seizure episodes (n = 4) terminated spontaneously. In the t1 group, 41.1% (*n* = 23) of the patients did not require intravenous treatment for their seizures, whereas the remaining 58.9% received intravenous treatment. The two groups showed statistically significant differences (*p* < 0.001) in the numbers of patients who experienced spontaneous termination of their seizures.

### Patients meeting t2 of SE demonstrated remarkably increased risk of mortality and unfavorable functional status at three months

At 3 months after SE onset, 23 (23.0%) patients died and 50 (50.0%) experienced seizure recurrence. Furthermore, 42 (42.0%) patients showed favorable outcomes (mRS: 0–2), while 58 (58.0%) had an unfavorable outcome (mRS: 3–6). The primary, secondary and tertiary outcomes are summarized in Table 3.

**Table 3:** Primary, secondary and tertiary outcomes at three months in total cohort

Outcome	T1 of SE (*n* = 56)	T2 of SE (*n* = 44)	Unadjusted RR (95%CI)	*P* value	Adjusted RR (95%CI)	*P* value
Mortality	6(10.7%)	17(38.6%)	3.606 (1.552–8.376)	0.003**	2.924^a^ (1.221–7.003)	0.016*
Recurrence	25(44.6%)	25(56.8%)	1.273 (0.862–1.878)	0.224	1.283^b^ (0.882–1.867)	0.193
mRS (3–6)	24(42.9%)	34(77.3%)	1.803 (1.280–2.539)	0.001**	1.664^c^ (1.184–2.340)	0.003**

Modified Poisson regression was used to evaluate the association of time points of SE with each outcome

*RR* Risk ratio, *CI* Confidence interval, *mRS* Modified Rankin Scale, *SE* Status epilepticus

^a^Adjusted for age, history of prior seizures and potentially fatal etiology

^b^Adjusted for history of prior seizures

^c^Adjusted for age, potentially fatal etiology and bilateral lesions/diffuse cerebral edema

*p<0.05, **p<0.01

Univariate analysis indicated that an increased risk of mortality was found if the patient had older age, a potentially fatal etiology and no history of prior seizures (*p* < 0.05). An unfavorable functional status was more likely if the patient had older age, a potentially fatal etiology and presented with bilateral lesions/diffuse cerebral edema (*p* < 0.05) (Tables 4 and 5).

**Table 4:** Univariate analyses of mortality at 3 months after SE onset

Variable	Total	Dead (*n* = 23)	Survivor (*n* = 77)	*P* value	OR (95%CI)
Age, median (IQR), years	66(53,75)	75(59,87)	65(47.5,71)	0.011*	–
Gender, male, No. (%)	61(61.0%)	12(52.2%)	49(63.6%)	0.340	0.623(0.243–1.636)
History of prior seizures, No. (%)	33(33.0%)	3(13.0%)	30(39.0%)	0.023*	0.235(0.070–0.85)
Acute symptomatic etiology, No. (%)	50(50.0%)	14(60.9%)	36(46.8%)	0.342	1.772(0.713–4.329)
Potentially fatal etiology, No. (%)	51(51.0%)	18(78.3%)	33(42.9%)	0.004**	4.8(1.679–12.6)
Bilateral lesions/diffuse cerebral edema, No. (%)	49(49.0%)	10(43.5%)	39(50.6%)	0.637	0.750(0.292–1.862)
Interictal epileptiform activity, No. (%)	39(39.0%)	7(30.4%)	32(41.6%)	0.466	0.615(0.242–1.596)

*IQR* Interquartile range, *mRS* Modified Rankin Scale, *OR* Odds ratio, *CI* Confidence interval

*p<0.05, **p<0.01

**Table 5:** Univariate analyses of functional status at 3 months after SE onset

Variable	Total	mRS 3–6 (*n* = 60)	mRS 0–2 (*n* = 40)	*P* value	OR (95%CI)
Age, median (IQR), years	66(53,75)	70(59.25,81.75)	61(37.5,67)	<0.001***	–
Gender, male, No. (%)	61(61.0%)	35(58.3%)	26(65%)	0.537	0.754(0.323–1.663)
History of prior seizures, No. (%)	33(33.0%)	17(28.3%)	16(40%)	0.279	0.593(0.253–1.384)
Acute symptomatic etiology, No. (%)	50(50.0%)	35(58.3%)	15(37.5%)	0.066	2.333(1–5.358)
Potentially fatal etiology, No. (%)	51(51.0%)	36(60.0%)	15(37.5%)	0.041*	2.5(1.068–2.771)
Bilateral lesions/diffuse cerebral edema, No. (%)	49(49.0%)	35(58.3%)	14(35.0%)	0.026*	2.6(1.105–2.131)
Interictal epileptiform activity, No. (%)	39(39.0%)	23(38.3%)	16(40.0%)	0.999	0.932(0.427–2.09)

*IQR* Interquartile range, *mRS* Modified Rankin Scale, *OR* Odds ratio, *CI* Confidence interval

*p<0.05, ***p<0.001

These variates were then entered into a modified Poisson regression model. In unadjusted analysis, patients meeting second time point criteria of SE demonstrated nearly four times the risk of mortality compared to those only reached first time point (RR 3.606, 95%CI 1.552–8.376, *p* = 0.003). After adjusting confounders, including age, history of prior seizures and potentially fatal etiology, patients meeting second time point criteria of SE was still associated with an increased risk of mortality (RR 2.924, 95%CI 1.221–7.003, *p* = 0.016).

In regard to functional status, when using unadjusted analysis, patients with first time point of SE were approximately two times more likely to have favorable functional status compared to those meeting second time point (RR 1.803, 95%CI 1.280–2.539, *p* = 0.001). Patients with first time point of SE was associated with an increased chance of good functional status after confounders (age and potentially fatal etiology) adjustment (RR 1.664, 95%CI 1.184–2.340, *p* = 0.003) (Table 3).

### Seizure recurrence rate at three months did not differ significantly between two cohorts

Patients were probably to have seizure recurrence after undergoing an episode of SE as fifty patients (50.0%) experienced at least another epileptic seizure at 3 months after SE onset. Patients who reached t2 of SE were more likely to experience seizure recurrence than those who only reached t1 (56.8, 44.6%). However, the recurrence rate did not differ significantly between the two cohorts, regardless of performing unadjusted or adjusted analysis in the overall (Table 3).

### Subgroup analysis of outcomes: different SE semiology (convulsive SE and focal SE with impaired consciousness)

Furthermore, we analyzed mortality and unfavorable functional status in patients with convulsive and focal SE with impaired consciousness, respectively, at two time points. Patients with convulsive SE who reached t2 demonstrated a remarkably increased risk of mortality (unadjusted analysis-RR 5.600, 95%CI 1.289–24.327, *p* = 0.022; adjusted analysis-RR 4.837, 95%CI 1.030–22.705, *p* = 0.046) and unfavorable functional status (unadjusted analysis-RR 2.182, 95%CI 1.270–3.750, *p* = 0.005; adjusted analysis-RR 2.121, 95%CI 1.192–3.774, *p* = 0.011) compared to those only reached first time point (Table 6). However, the mortality and rate of unfavorable functional status did not differ significantly between the two cohorts in focal SE patients with impaired consciousness (Supplementary Table 1). We also analyzed seizure recurrence rates in patients with convulsive and focal SE, respectively, however there were no significant differences (Table 6 & Supplementary Table 1).

**Table 6:** Primary, secondary and tertiary outcomes at 3 months in convulsive SE (CSE)

Outcome	T1 of SE (*n* = 32)	T2 of SE (*n* = 20)	Unadjusted RR (95%CI)	*P* value	Adjusted RR (95%CI)	*P* value
Mortality	2(6.3%)	7(35.0%)	5.600 (1.289–24.327)	0.022*	4.837 (1.030–22.705)	0.046*
Recurrence	16(50.0%)	10(50.0%)	1.000 (0.572–1.748)	1.000	0.988 (0.573–1.703)	0.965
mRS (3–6)	11(34.4%)	14(70.0%)	2.182 (1.270–3.750)	0.005**	2.121 (1.192–3.774)	0.011*

Modified Poisson regression was used to evaluate the association of different time points of convulsive SE with each outcome

*RR* Risk ratio, *CI* Confidence interval, *mRS* Modified Rankin Scale, *SE* Status epilepticus

^a^ Adjusted for age and potentially fatal etiology

^b^ Adjusted for history of prior seizures

^c^ Adjusted for age, history of prior seizures and potentially fatal etiology

*p<0.05, **p<0.01

In addition, we have performed ROC curve to find better seizure duration time cut-offs to discriminate between good and bad prognosis. The results showed that the AUC for mortality is 0.789 (95%CI: 0.650–0.929), and 0.741 (95%CI: 0.603–0.878) for unfavorable functional status in convulsive SE, of which the best cut-off is 17.5 min. We have also conducted ROC curve in focal SE, however none of the analyses yielded statistically significant results (Supplementary Fig. 1).

## Discussion

To our knowledge, this is the first prospective cohort study to describe the clinical characteristics and prognoses of SE based on the new conceptual definition proposed by the ILAE in 2015. We found significant differences in seizure etiology, seizure termination, and outcomes between the two groups. The study indicated that prolonged seizure duration tends to have a remarkably increased risk of mortality and unfavorable functional status, yet seizure recurrence did not differ significantly. Several aspects of our results deserve further attention.

First, we found that episodes of SE reaching t2 were more common among patients with acute symptomatic process. This reflects the fact that acute symptomatic seizures tend to have a long duration. Acute symptomatic seizure is a clinical seizure occurring at the time of a systemic insult or in close temporal association with a documented brain insult [27]. This coincides with previous knowledge that the majority of prolonged cases of SE are due to acute symptomatic causes [28], which tend to be associated with higher rates of morbidity and mortality than chronic etiologies [29, 30]. The underlying etiology of SE often influences the risk of mortality [31]. Only 33.0% of patients with SE have had previous seizures. Our results indicated that the most common causes of SE were remote cerebrovascular disease, brain injury, acute cerebrovascular disease, drug reduction/withdraw, poor compliance and acute CNS infection. Specific causes seem to vary among different populations because methodological variability among studies is high and limits direct comparisons [32].

Second, unadjusted and adjusted analyses of correlations between SE timing and outcomes revealed that patients meeting second time point of SE demonstrated a remarkably increased risk of mortality and unfavorable functional status at 3 months compared to those only reached the first time point. To date, age, seizure etiology, and seizure duration have been used as independent predictors of SE, with seizure duration often being the only modifiable risk factor through timely management [33, 34]. One study demonstrated that the estimated RR between the group of patients whose SE lasted ≥1 h and the group of those whose SE lasted < 1 h was 9.79 [8]. We used a cut-off time of 5 min for convulsive seizures and received similar results.

It is known that underlying etiology is a more important determinant of outcome than SE itself, and PFE was the most predictive factor for mortality [20]. Univariate analysis did confirm that a potentially fatal etiology correlates with mortality and an unfavorable functional status. A previously unrecognized finding is that SE timing is still significant after adjusting these covariates. Another interesting point in this context is that patients with history of prior seizures could be associated with better outcome, which may relate to shorter seizure duration time and underlying cause of drug reduction/withdraw and poor compliance in these patients.

Third, it has been reported before that SE increased the risk for subsequent unprovoked seizure by 3.3 times (95% CI 1.8–6.1) compared with brief acute symptomatic seizures [35]. We discovered a high seizure recurrence rate regardless of no significant difference between the two cohorts of SE, probably because that we analyzed seizure recurrences both in the setting of a persisting or reemerging acute symptomatic cause and in the setting of an unprovoked seizure [36].

The prognosis of SE has long been considered as poor. This was likely due to the high frequency of comorbidities such as stroke and other forms of brain injury and the reduced ability of the patients with SE to tolerate the extreme metabolic stress placed on the brain and the body. In most cases, SE is an epiphenomenon of severe brain injury rather than a primary offender [37]. It is a marker of injury severity. Taken together, these results suggest that earlier recognition and treatment of SE and its underlying causes can help ensure favorable outcomes.

Fourth, there were significant differences in short-term outcomes for patients with CSE between these two cohorts. However, among patients with focal SE, there were no significant differences in the outcomes. We have also performed ROC curve to find better seizure duration time cut-offs to discriminate between good and bad prognosis. The results showed that the best cut-off is 17.5 min in convulsive SE, while no statistically significant results were found in focal SE. The setting of t1 at 5 min and t2 at 30 min in convulsive SE was based on previous animal experiments and clinical research. However, there is limited information available to define t1 and t2 in focal SE. The evidence is incomplete and there is considerable variation; therefore, these time points should be considered as the best estimates currently available. Previous studies showed obvious differences in time point settings for SE, and only a few studies [38–40] chose the cut-off of 5 min in adults with convulsive SE. Therefore, it is important to emphasize that the proposed time points are merely a framework and must not be treated as a doctrine, but reflect our current knowledge of SE. Future advances in basic, epidemiologic, and clinical research will undoubtedly lead to modifications and major revisions of this proposed definition of SE.

This revised definition of SE builds on the recognition that rapid initiation of treatment is paramount in patients with prolonged seizure activity. As shown in our study, prolonged seizure duration was associated with a remarkably increased risk of mortality and unfavorable functional status. Furthermore, seizure duration is the only modifiable prognostic factor that can be improved by expeditiously administering antiepileptic medications [41]. Additionally, prolonged seizures may lead to changes in the composition and location of gamma-aminobutyric acid A receptors and N-methyl-D-aspartic acid receptors, leading to loss of inhibition and increased excitation, leading to progressive resistance to benzodiazepines (BZDs) in animal models, which are apparent in specific animal models with progressive resistance to BZDs with long seizures [42]. Despite the progress in basic science, translating the findings to the clinical field remains difficult. The American Epilepsy Society has already published a guideline on the treatment of SE [43], yet we found pervasive, substantial gaps between recommended and real-world practice with regard to timing, dosing, and sequence of antiepileptic therapy. The short time window for SE usually requires immediate management, while some patients are still in prehospital settings. Lack of the ability of symptom recognition and rapid treatment initiation of emergency medical service personnel and patients’ caretakers adds to the difficulty in clinical practice. However, we should at least accomplish the best management in hospital settings.

### Limitations

Our study has some limitations that need to be considered. First, the sample size was relatively small and included subjects from a single tertiary medical center, with a disorder as heterogenic as SE. The tertiary hospital setting may also confer a selection bias. Indeed, this may have resulted in the inclusion of more patients with severe SE. Although the data were homogenous with comparable faculty and treatment, the results may not be extrapolated to other settings or populations. Second, in our study, seizure duration time was assessed by clinical manifestations. Continuous EEG monitoring is a good way to exactly define the end of the seizure episode. However, ictal EEG monitoring may not be available in each case as some of the SE episodes occurred prehospital and the median seizure duration time was 30 min (IQR: 10-120 min) in our study, which was not long enough for the EEG preparation. Third, we analyzed the follow-up data at 3 months after SE onset, which represented the short-term outcomes. Part of the follow-up information on patient outcomes was obtained through telephone interviews, which might have resulted in some small biases. Fourth, confounders not considered in our study may have substantially influenced short-term outcomes, such as therapeutic decisions (selection and dosing of intravenous antiepileptic drugs), thus introducing the risk of bias into our results.

## Conclusions

Our study provides strong support for the new definition of SE, as previous evidence is incomplete and were mostly based on animal studies. Our findings suggest that prolonged seizure duration tends to have a remarkably increased risk of mortality and unfavorable functional outcome; however, seizure recurrence did not differ significantly between the two cohorts. Therefore, the time points proposed by the ILAE in 2015 should be considered the best estimates currently available. SE is a life-threatening and time-sensitive emergency that requires immediate treatment. While “time is brain” has traditionally described the pathophysiology of stroke, our current understanding of the SE reaffirms this mantra. Physicians must be educated about prompt recognition and appropriate management of SE.

## Supplementary Information

**Additional file 1: Supplementary Table 1.** Primary, secondary and tertiary outcomes at 3 months in focal SE. **Supplementary Fig. 1.** ROC curve for predicting mortality and unfavorable functional status in convulsive SE using seizure duration time. Mortality: AUC = 0.789, 95%CI: 0.650–0.929; unfavorable functional status: AUC = 0.741, 95%CI: 0.603–0.878; the best cut-off: 17.5 min.

## Data Availability

The datasets used and/or analyzed during the current study are available from the corresponding author on reasonable request.

## Abbreviations

SE: Status epilepticus
CNS: Central nervous system
BZD: Benzodiazepines
ILAE: International League Against Epilepsy
EEG: Electroencephalogram
NCSE: Nonconvulsive status epilepticus
EMSE: Epidemiology-based Mortality Score in Status Epilepticus
PFE: Potentially fatal etiology
CT: Computerized tomography
MRI: magnetic resonance imaging
mRS: Modified Rankin Scale
CI: Confidence interval
IQR: Interquartile range
AOR: Adjusted odds ratio
