# Supplementary material for: Timing matters: there are significant differences in short-term outcomes between two time points of status epilepticus
Source: BMC Neurol. 2022 Sep 14;22:348. doi: 10.1186/s12883-022-02868-y (PMC9472412; doi:10.1186/s12883-022-02868-y)
Supplement: Supplementary file 1 — Additional file 1: Supplementary Table 1. Primary, secondary and tertiary outcomes at 3 months in focal SE. Supplementary Fig. 1. ROC curve for predicting mortality and unfavorable functional status in convulsive SE using seizure duration time. Mortality: AUC = 0.789, 95%CI: 0.650–0.929; unfavorable functional status: AUC = 0.741, 95%CI: 0.603–0.878; the best cut-off: 17.5 min. [file 12883_2022_2868_MOESM1_ESM.docx]

**Supplementary Table 1** Primary, secondary and tertiary outcomes at 3 months in focal SE

| Outcome | T1 of SE  (n=24) | T2 of SE  (n=24) | Unadjusted RR（95%CI） | P  value | Adjusted RR  (95%CI) | P  value |
| --- | --- | --- | --- | --- | --- | --- |
| Mortality | 4(16.7%) | 10(41.7%) | 2.500  (0.909-6.879) | 0.076 | 1.372  (0.949-1.983) | 0.093 |
| Recurrence | 9(37.5%) | 15(62.5%) | 1.667  (0.913-3.044) | 0.096 | 1.805  (1.010-3.227) | 0.046* |
| mRS (3-6) | 13(54.2%) | 20(83.3%) | 1.429  (0.974-2.094) | 0.068 | 2.175  (0.817-5.792) | 0.120 |

Modified Poisson regression was used to evaluate the association of different time points of focal SE with impaired consciousness with each outcome.

^a^ Adjusted for potentially fatal etiology

^b^ Adjusted for history of prior seizures

^c^ Adjusted for age and potentially fatal etiology

RR: risk ratio, CI: confidence interval, mRS: modified Rankin Scale, SE: status epilepticus.

*p＜0.05

**Supplementary Figure 1**

**Figure 1A**

**Figure 1B**

ROC curve for predicting mortality and unfavorable functional status in convulsive SE using seizure duration time. Mortality: AUC=0.789, 95%CI: 0.650-0.929; unfavorable functional status: AUC=0.741, 95%CI: 0.603-0.878; the best cut-off: 17.5min.

ROC: receiver operating characteristic curve, AUC: area under curve, CI: confidence interval, SE: status epilepticus.
